# Cytotoxicity Profiling of *Annona Squamosa* in Cancer Cell Lines

**DOI:** 10.31557/APJCP.2019.20.9.2831

**Published:** 2019

**Authors:** Biba Vikas, Sukumaran Anil, P Remani

**Affiliations:** 1 *Regional Cancer Centre, Thiruvananthapuram, India, *; 2 *Department of Dentistry, Hamad Medical Corporation, Doha, Qatar. *

**Keywords:** Anticancer, cytotoxicity, Annona squamosal

## Abstract

**Objective:**

In the study our aim was to evaluate the cytotoxic activity of different solvent extracts of *Annona squamosa* seeds.

**Methods and materials:**

The four extracts used were petroleum ether, chloroform, ethyl acetate and methanol were tested using cytotoxicity assays.

**Results:**

Among the four extracts tested petroleum ether showed maximum cytotoxicity against a panel of cancer cell lines such as nasopharyngeal cancer (KB) cells, lung cancer (A-549) cells, breast cancer (MCF- 7) cells, leukemic (K-562) cells and inhibited the growth of murine cancer cells such as Dalton’s lymphoma ascites (DLA) and Ehrlich ascites carcinoma (EAC).

**Conclusion:**

Petroleum ether extract of *Annona squamosa* seeds showed cytotoxicity towards a panel of cancer cells meanwhile non-significant cytotoxicity towards normal cells.

## Introduction

Natural products such as plants, animals and minerals have been the basis of treatment of human diseases. The study of ethnopharmacology and traditional medicine leads numerous drugs to international pharmacopoeia. Traditional medicines can lead to drug design and possible targets for scientific analysis. Powerful new technologies are available for the medicinal plants screening are revolutionizing drug discovery. By looking at the historical trends in drug and medical developments, it helps to understand how current drug development will benefit. Throughout the history of drug development plants are an important source for the discovery of novel bioactive compounds. Many compounds have been derived from the plant species for the treatment of a number of ailments. The R and D thrust in the pharmaceutical sector is focused on development of new drugs, innovative/indigenous processes for known drugs and development of plant-based drugs through investigation of leads from the traditional systems of medicine. In addition, many nutraceuticals are being consumed from unregulated markets for their perceived benefits in health care and improvement of quality of life. Natural pharmaceuticals, nutraceuticals and cosmeceuticals are of great importance as a reservoir of chemical diversity aimed at new drug discovery and can be explored as potential anticancer drugs. Today, there are at least 120 distinct chemical substances derived from plants that are considered important drugs and are currently in use in one or more countries in the world. Some of these drugs are simply a chemical or chemicals extracted from plant materials and put into a capsule, tablet, or liquid. 

While many drugs have originated from biologically active plant chemicals, and many other medicinal uses of plants can be attributed to various active chemicals found in them, there is a distinct difference between using a medicinal plant and a chemical drug. Drugs usually consist of a single chemical, whereas medicinal plants can contain 400 or more chemicals. It’s relatively easy to figure out the activity and side effects of a single chemical, but there is just no way scientists can map all the complex interactions and synergies that might be taking place between all the various chemicals found in a plant, or a traditionally prepared crude plant extract, containing all these chemicals. It is not unusual that plant contain a single chemical which is a carcinogen and also maybe five other chemicals that are anticancerous and which may counteract with the “*bad*” chemical. Overall, the plant extract may even provide some type of anticancerous effect. There are common features for herbal medicines: Herbal medicines are different from clinically defined medicines in their character as well as in their medicinal value. They are based mostly on herbal products. Usually, they are multidrug formulations including animal and mineral products as essential components or additives. In herbal therapy, data on pre-clinical investigations are often incomplete although in majority of cases the therapeutic experiences have been accumulated over centuries. Some of them follow practices based on, for example, mistaken beliefs, faulty experimentation, or inaccurate information that can be dangerous. They mostly include empirically defined doses and course of medication. The identity of plant species used is often controversial. Safety measures are poorly adopted, in most cases. Additives are frequently used; many of them also have therapeutic actions. The overall goal in drug development is quality, safety and efficacy. 

All measures in drug development are directed to this goal. The requirements of health authorities on quality, safety and efficacy are standardized on a high level based on the development procedure for the herbal as well as synthetic drugs. Health authorities are reluctant to accept traditional drug preparations from other cultural areas without well-documented data on quality, safety and efficacy. In many developing countries, appropriate utilization of local resources to cover drug needs is dependent on preliminary scientific study to determine the efficacy and safety of the preparations based on plant drugs that are used on an empirical basis in traditional medicine.

Despite many shortcomings, the number of users of herbal drugs is increasing in the developing as well as the industrialized world. Traditional herbal medicines, although currently serving the health care needs of majority of the world’s population, can be further increased in coverage and broadened in terms of safety and efficacy provided that some basic principles of drug preparation, evaluation and uses are brought into practice. The message is clear that phytotherapy acts as a bridge between traditional medicine and modern medicine. The developments of plant derived drugs have always been a multi-step procedure starting with a crude extract followed by the standardized extract and ending up with isolated constituents. Quite often sufficient quality control and drug standardization is lacking for traditional recipes. Ethno pharmacological leads have resulted in the introduction of new single molecule drugs but have a greater role to play if crude extracts are accepted for clinical use. Colorimetric MTT assay in 96 well plates were developed, which was adopted in the screening system in the NCI, based on a new idea that is disease-oriented screening (DOS) using about 60 human tumor cell lines (Tashiro et al., 1992). Some of the important plant products used in chemotherapy are based on this type of screening and finally marketed as drugs. There were reports that Annonaceae plants have been used in Indian system of medicine for diseases having symptoms similar to cancer. In this study our aim was to evaluate the cytotoxic activity of different solvent extracts of *Annona squamosa* seeds belonging to the Annonaceae family.

## Materials and Methods

*Materials*

*Plant*

The seeds of *Annona squamosa* (AS) were collected from Thiruvananthapuram district, Kerala State authenticated by the taxonomist and a voucher specimen TBGT 57051 have been kept in the herbarium of Tropical Botanical Garden, Research Institute, Thiruvananthapuram. The shade dried and pulverized seeds were used for soxhlet extraction in a soxhlet apparatus using petroleum ether (ASPE), chloroform (ASCH), ethyl acetate (ASEA) and methanol (ASME) as the solvents.

*Tumor cell lines*

Nasopharyngeal cancer (KB) cells, Lung cancer (A-549) cells, Breast cancer (MCF-7) cells, Leukemic (K-562) cells were obtained from National Centre for Cell Sciences, Pune. The murine cancer cells Dalton’s lymphoma ascites (DLA) and Ehrlich ascites carcinoma (EAC) were propagated in the peritoneal cavity of Balb/C mice.

*Media and Chemicals*

Various chemicals were procured from different sources as stated below: Dulbecco’s modified eagle’s medium (DMEM) and antibiotics, streptomycin, penicillin, Amphotericin-B from Hi- Media Laboratories, Mumbai. Fetal Calf Serum, Trypsin, Hoechst stain and MTT (3-(4, 5- dimethyl thiazol 2-yl)-2, 5-diphenyl tetrazolium bromide) from Sigma Chemical Company, USA. All other chemicals, solvents and reagents used were of analytical grade.

*Sterilization of Glass wares*

All glass wares and filtration apparatus used for cell culture were soaked in a solution of sterinol overnight, cleaned using brush and washed thoroughly under running water. They were then boiled for one hour and rinsed in distilled water and dried in a hot air oven. These were then autoclaved at a pressure of 15 lbs for 15 min, dried and used for experiments.

**Figure 1:**
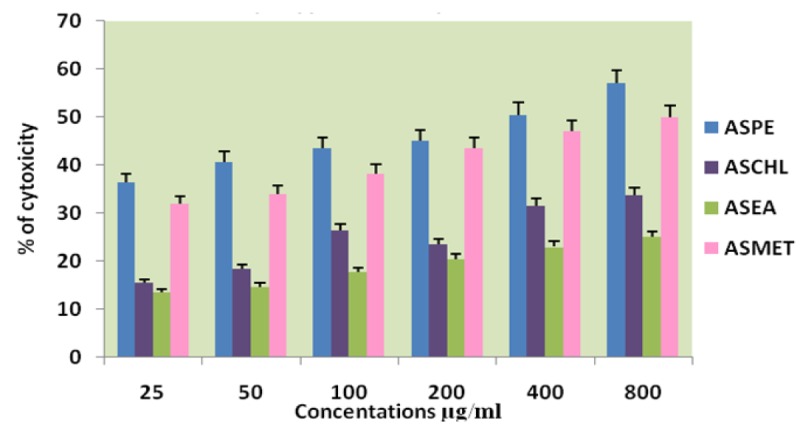
Effects of Different Solvent Extracts on DLA Cells Trypan Blue Dye Exclusion Method

**Figure 2:**
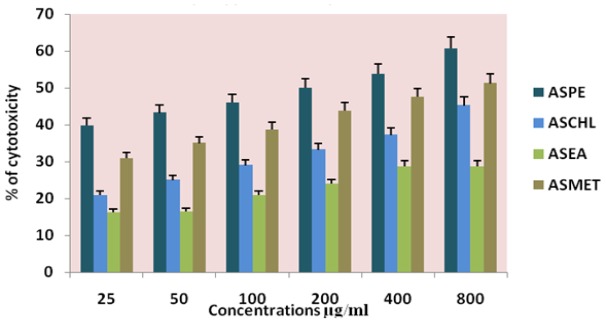
Effects of Different Solvent Extracts on K-562 Cells Trypan Blue Dye Exclusion Method

**Figure 3:**
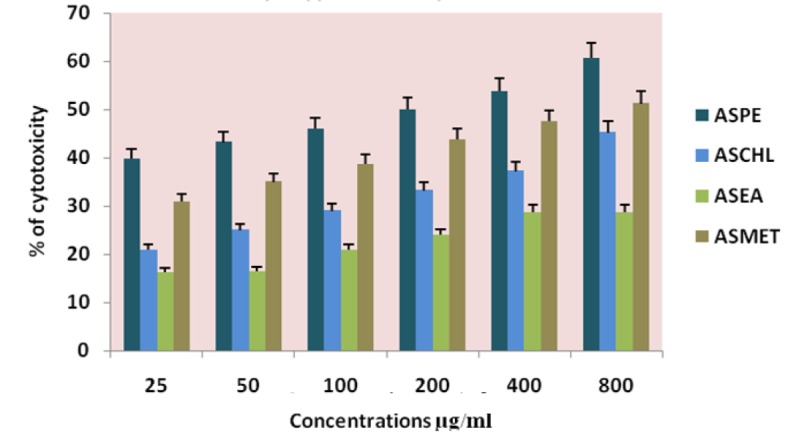
Cytotoxicity of *Annona Squamosa* Seed Extracts on KB Cells at 24 Hours by Mttassay

**Figure 4:**
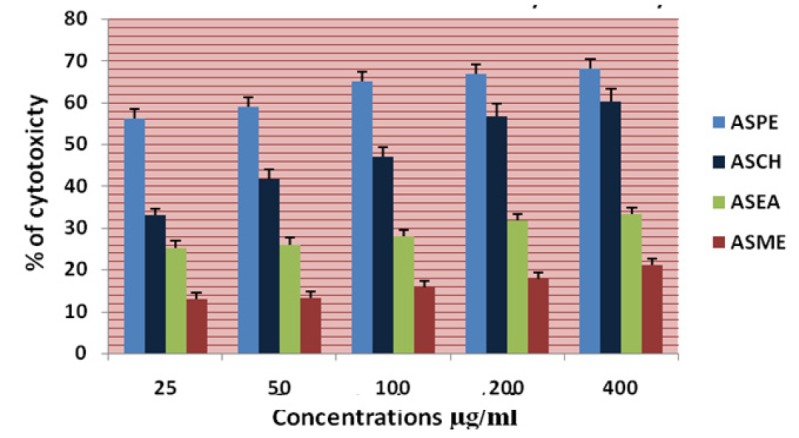
Cytotoxicity of *Annona squamosa* Seed Extracts on A-549 Cells at 24 Hours by MTT Assay

**Figure 5:**
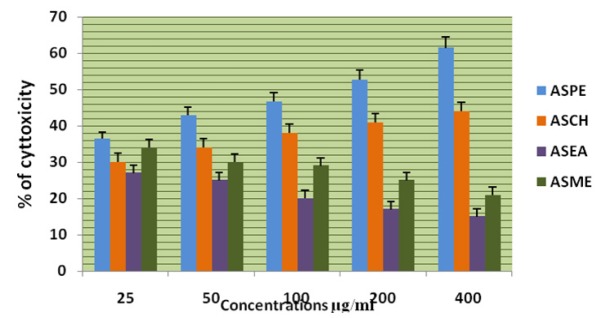
Cytotoxicity of *Annona Squamosa* Seed Extracts on MCF-7 Cells at 24 Hours by MTT Assay

**Figure 6:**
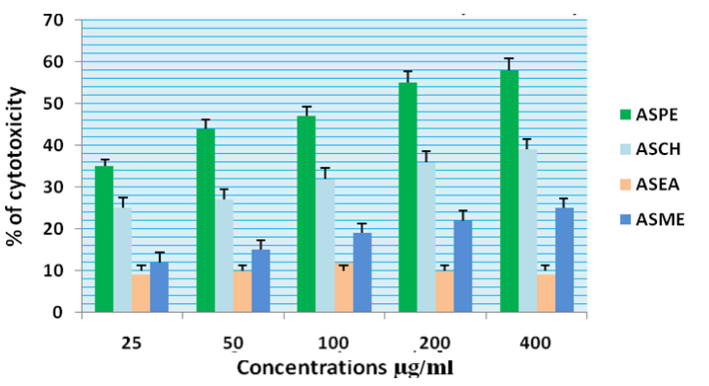
Cytotoxicity of *Annona Squamosa *Seed Extracts on K-562 Cells at 24 Hours by MTT Assay

**Figure 7:**
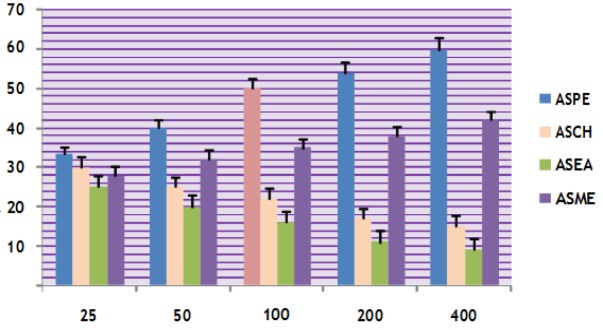
Cytotoxicity of *Annona Squamosa* Seed Extracts DLA at 24 Hours by MTT Assay

**Figure 8:**
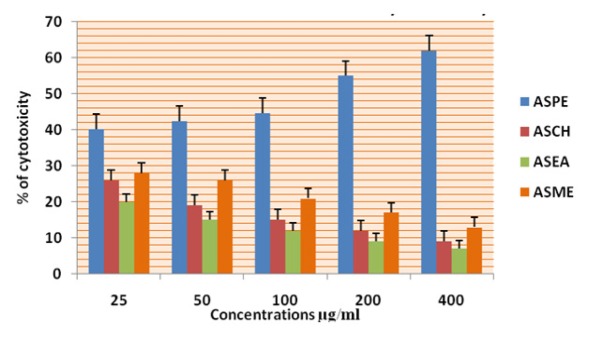
Cytotoxicity of *Annona Squamosa* Seed Extracts EAC at 24 Hours by MTT Assay

**Figure 9:**
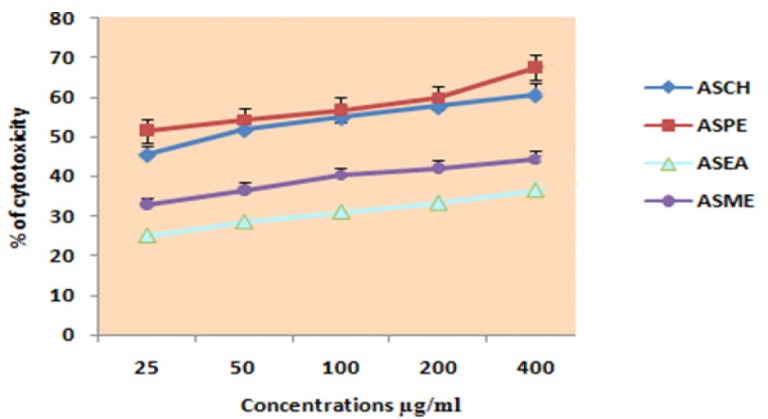
Cytotoxicity of *Annona Squamosa* Seed Extracts KB at 24 Hours by MTT Assay

**Figure 10:**
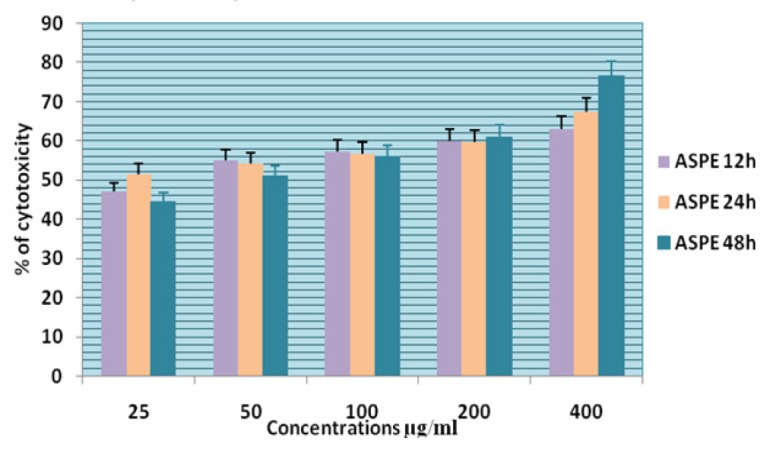
Cytotoxicity of ASPE on KB Cells at 12,24 & 48 Hour by MTT Assay

**Figure 11:**
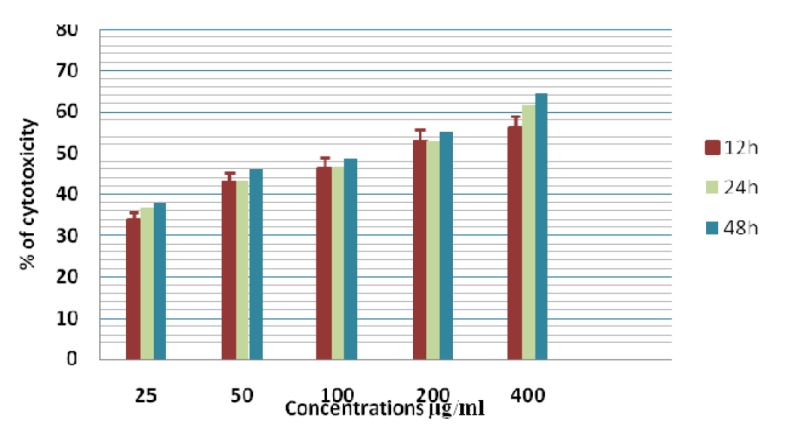
Cytotoxicity of ASPE on A-549 Cells at 12,24 & 48 Hours by MTT Assay

**Figure 12:**
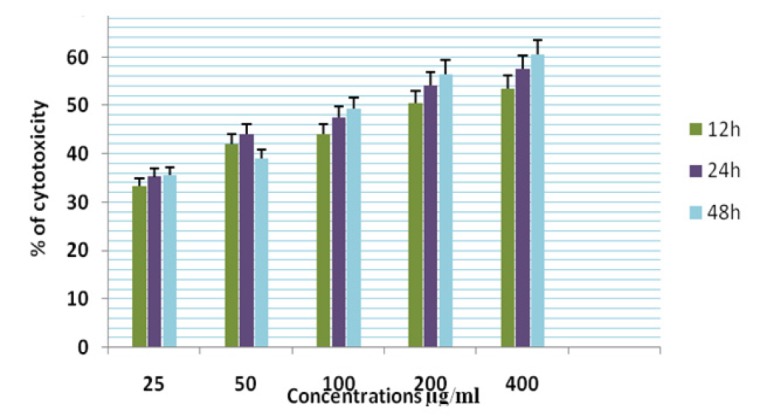
Cytotoxicity of ASPE on MCF-7 Cells at 12,24 & 48 Hours by MTT Assay

**Figure 13:**
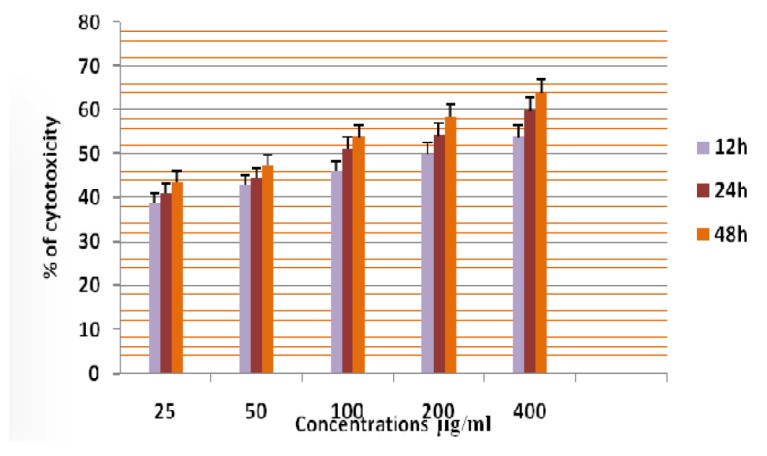
Cytotoxicity of ASPE on K-562 Cells at 12,24 & 48 Hours by MTT Assay

**Figure 14:**
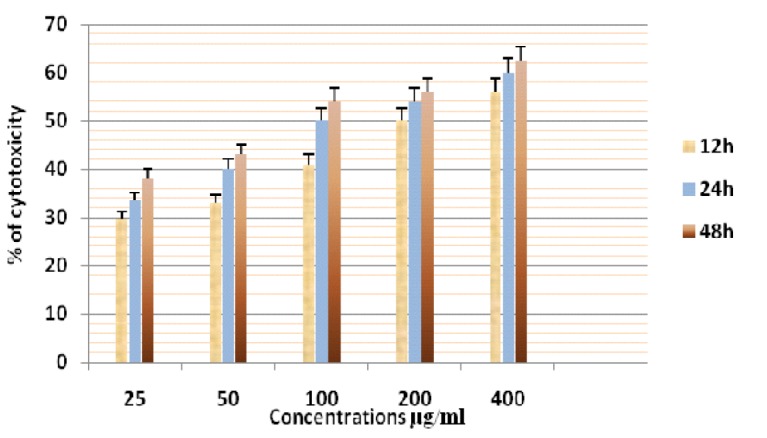
Cytotoxicity of ASPE on DLA Cells at 12,24 & 48 Hours by MTT Assay

**Figure 15:**
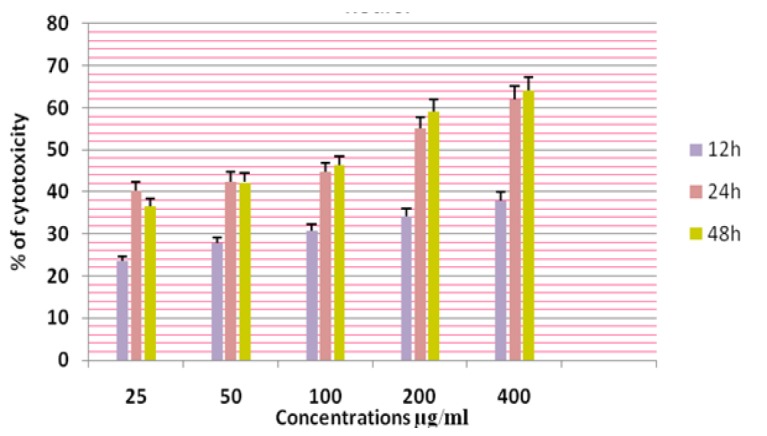
Cytotoxicity of ASPE on EAC Cells at 12,24 & 48 Hours by MTT Assay

**Figure 16:**
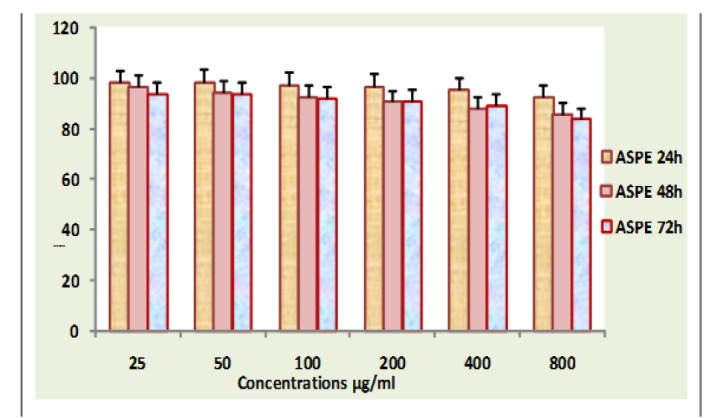
Lymphocyte Viability Assay

**Table 1:** IC_50_ Values of *Annona Squamosa* Petroleum Ether Extract

Sl.No.	Cell line	IC _50_ values (µg/ml) ASPE
1	KB	22.4 µg/ml
2	A-549	154 µg/ml
3	MCF-7	136.1 µg/ml
4	K-562	90.7 µg/ml
5	DLA	149 µg/ml
6	EAC	98 µg/ml

*Methodology*

*Solvent Extraction*

The organic extracts of *Annona squamosa* seeds have been prepared as follows: The seeds were subjected to exhaustive solvent extraction using a soxhlet apparatus with a panel of solvents, Petroleum ether (BP 60-80°C), Chloroform (BP 61- 62°C), Ethyl acetate (BP 77.1°C) and Methanol (BP 64.5-65.5°C). As per the standard soxhlet procedure, the powder obtained was packed in a porous cellulose thimble. The thimble was placed in an extraction chamber, which was suspended above a flask containing the solvent and below a condenser. As the flask is heated to the boiling point of the solvent, solvent gets evaporated and moves up into the condenser, where it gets condensed and is collected in the extraction chamber. Seed powder in the extraction chamber gets extracted with the hot solvent. Due to the peculiar design of the apparatus, capillary force draws the entire solvent from the extraction chamber back to the original round bottom flask. Several cycles of extraction were underwent such cycles allows complete isolation of the extractable compounds of that particular solvent. At the end of the extraction process, solvent containing extractable portion is removed from the flask. The same powder is then subjected to sequential extraction with the solvent of higher polarity from the panel of selected solvents.The extracts were then concentrated by using Rotavapour. The *Annona squamosa* seed extracts prepared using petroleum ether, chloroform, ethyl acetate and methanol were termed as ASPE, ASCH, ASEA and ASME respectively. Stock solutions of the concentrated extracts were prepared as 20mg/ml in DMSO for analyzing its in vitro cytotoxicity.

*Preparation of culture media*

Culture media was prepared by mixing DMEM powder (Glucose, Lglutamine, pyridoxine, HCl, without pyruvate) in autoclaved triple distilled water. To this 1.95gm of HEPES buffer and 3.75gm sodium bicarbonate was added. Antibiotics such as Penicillin (500 µl) and Streptomycin (500 µl) and fungicide- amphotericin-B (750 µl) were also added. The volume was then made up to 1,000 ml and the pH was adjusted to 7.2 - 7.4. The medium was then filtered under negative pressure using 0.22 pm cellulose filter. Sterility of the medium was tested before 10% FCS was mixed with the medium before use for culture.

*Maintenance of adherent cell lines*

Adherent cell lines will grow in vitro until they form a monolayer over surface area available or medium depleted of nutrients. Adherent cells KB, A-549, and MCF-7 were cultured in tissue culture flasks. The cells were disaggregated by trypsinization and subcultured when the monolayer reached about 70% confluency. Cells were also cryopreserved at -80^o^C.

*Procedure*

With an inverted microscope degree of confluency of the cell monolayer was assessed and the absence of bacterial and fungal contaminants was confirmed. Spent medium was removed. Cells were washed with PBS-EDTA for removing all the traces of serum. Trypsin/EDTA (500 ml) was applied onto the cell monolayer, and the flask was swirled to cover the monolayer with trypsin. Flasks were incubated at 37^o^C for 2-3 minutes. The flasks were examined under the inverted microscope to ensure uniform detachment of the cells. 1-2 ml of medium was added to the flask as fast as possible to lessen the trypsin induced stress, and the contents of the flask were transferred to a centrifuge tube. Cells were centrifuged at 1,500 rpm, for 10 minutes. The supernatant was discarded, and the cells were resuspended in minimum volume of medium. Cells were counted using a Haemocytometer and the required numbers of cells were sub cultured to a new flask containing fresh DMEM with 10% FCS. Repeat this process as demanded by the growth characteristics of the cell line.

*Maintenance of suspension cell lines*

In general terms cultures derived from blood (eg. lymphocytes grow in suspension) cells may be seen as single cells or clumps. For these types of cell lines sub culturing is done by dilution in small volume of media before counting. The culture was viewed using an inverted phase contrast microscope; cells growing in exponential phase should be bright, round and refractive. The cell suspension was mixed well and dispersed uniformly by repeated pipetting in order to make single cell suspension. The cells were counted and 1x10^6^ cells were seeded to fresh bottle containing 10 ml of DMEM medium with 10% FBS, antibiotics and incubated at 37°C and sub cultured every third day. On the day of the experiment, single cell suspension was prepared. The cells were counted, viability was checked and concentration was adjusted with medium containing 10% FBS and the antibiotics.

*Establishing Cell Cultures from Frozen Cells*

10 ml of growth medium was placed in a 15-ml conical tube. Thawed the frozen cryovial of cells within 40-60 seconds by gentle agitation in a 37°C water bath. Removed the cryovial from the water bath and decontaminated the cryovial by immersing it in 70% ethanol at RT (Room Temperature). Transferred the thawed cell suspension to the conical tube containing 10 ml of growth medium. Cells were collected by centrifugation at 200 x g for 5 minutes at RT. Removed the growth medium by aspiration. Resuspended the cells in the conical tube in 5 ml of fresh growth medium. Added 10 ml of growth medium to a 75-mm tissue culture flask and transferred the 5 ml of cell suspension to the same tissue culture flask. Placed the cells in a 37°C incubator at 5% CO_2_. Monitored cell density daily. Cells were passaged when the culture was at 50% confluency.

*Determination of cell viability.*

*Cell viability Determination by Trypan Blue Exclusion Method*

*Overview*

Trypan blue is a dye that is used to determine the viability of a cell. Living cells exclude the dye, whereas dead cells will take up the blue dye. The blue stain is easily visible, and cells can be counted using a light microscope. The reactivity is negatively charges and doesn’t interact with the cell unless the membrane is damaged. Therefore, all the cells that exclude the dye are viable. When the cells are confluent, remove the cell media through aspiration and add 5ml of PBS swirl and aspirate. Then add 2ml of Trypsin/ EDTA and swirl to cover the monolayer of cells. Incubate for few minutes at 37°C. To remove the cells, strike the side of the plate or the flask with the palm. Check under a microscope to ensure that all the cells are dislodged. Add 8ml of cell media containing fetal calf serum (FCS) to the cells. The FCS was neutralized the action of trypsin. Transfer the cell suspension to a sterile centrifuge tube.

Centrifuge the cell suspension at 1,500 rpm to pellet the cells. Wash the cell pellet with PBS and repeat the centrifugation. Resuspend the cell pellet in appropriate volume of PBS or cell media, depending on the subsequent use of the cells. Dilute 101 of cell suspension and place 10µl on a haemocytometer (between the counting slide and glass coverslip). Count the cells under a microscope. There are grid markings on the haemocytometer that can be seen under magnification. Count the cells in all four other quadrants of the grid. Divide this number by four to determine the average number of cells in one quadrant. To calculate the number of cells, multiply the average number of cells per quadrant by dilution factor. Multiply this number by 10,000 to calculate the number of cells in one ml of suspension. To calculate the total number of cells, multiply the number of cells per ml by the volume (ml) of the cell suspension.

*Calculating the % of viable cells*

The cells (10,000) are suspended in 500 µl media. Treated with drug at varying concentration. Incubate for 24hrs. Centrifuge at 1,500rpm for 10 minutes. Discard 400 µl medium. Resuspended the pellet in the remaining medium. Mix 0.5mg of Trypan blue in 1 ml PBS. Take 10 µl of cell suspension and mix with trypan blue solution. Incubate for 5minutes at room temperature. Count the numbers of unstained cells on the haemocytometer under a microscope. As mentioned above dead cells will take up the trypan blue stain. First count blue cells in the field and then white cells. Count the total number of cells. Determine the % of viable cells by dividing the number of unstained cells by the total number of cells and multiplying by 100.The equation is as follows: % of Cytotoxicity = (No. of blue cells/Total no. of cells) X 100.

*Evaluation of cytotoxicity using MTT Assay*

The assay detects living, but not dead cells and the signal generated is dependent on the degree of activation of the cells. This method can therefore be used to measure cytotoxicity, proliferation or activation (Mosmann, 1983). The chief advantage of this assay is that it requires fewer cells than standard cytotoxicity assays. In addition, it allows for multiple sample concentrations on a single 96-well plate which is then rapidly quantitated using an automated spectrophotometric microplate reader. Moreover, it is rapid, precise and does not involve any radioisotope. MTT[3-(4,5Dimethylthiazol-2yl)-2,5-Diphenyl Tetrazolium Bromide] assay, is based on the mitochondrial dehydrogenase enzyme from viable cells to cleave the tetrazolium rings of the pale yellow MTT and form a dark blue formazan crystal which is largely impermeable to cell membranes, thus resulting in its accumulation within healthy cells.

Solubilisation of the cells by addition of a detergent results in the liberation of the crystals, which are solubilised. The number of surviving cells is directly proportional to the level of formazan product created. The colour can be quantified using a multiwell plate reader. The in vitro response of *Annona squamosa* extracts (against tumour cell lines were studied using MTT assay.The cell lines were maintained in DMEM medium, supplemented with 10% FCS. Briefly, cells in the log phase of growth were harvested, counted and seeded (5x10^3^ cells/well in 100 µl) in 96 well titre plates (Axygen) and PBS were added to the outer wells 

(200 µl/well). After 24 hours of incubation at 37°C in 5% CO_2_ to allow cell attachment, media were removed; cultures were treated with various concentration of ASPE (50-800 µg/ml) diluted with medium. The negative controls were also kept (cells and media). The plates were further incubated for 24, 48 and 72 hours. On completion of incubation, with the extract, media was removed without disturbing the adherent cells. In the case of suspension cells lines, the media was removed after the plates were centrifuged at 2,000 rpm for 15 minutes. To each well, 100 µl of 5mg/ml stock solution of MTT was added and plates were further incubated for 2 hours in dark at 37°C in a CO_2_ incubator. 100 µl of lysis buffer was added to each well and the plates were further incubated for 4 hours in dark in a CO_2_ incubator and absorbance was read using ELISA plate reader. Three replicates were set up for each concentration. The concentration required to reduce absorbance by 50% (IC_50_) in comparison to control cells were determined. Percentage of Growth Inhibition =100 - absorbance of treated cells x 100/absorbance of control cells.

*Isolation of lymphocytes from whole blood*

*Principle*

In 1968, Boyum described methods for the isolation of mononuclear cells from circulating blood and bone marrow. The solution contains Ficoll and Sodium Diatrizoate, adjusted to a density of 1.077± 0.001. This medium facilitates rapid recovery of viable lymphocytes from small volumes of blood on centrifugation. It is usually employed as the initial isolation step prior to enumeration of T, B and null lymphocytes. In brief, 3ml of blood was taken in heparinised test tube.5ml of PBS was added and mixed well by inversion. 3ml of ficoll hypaque solution was added in a conical centrifuge tube. Carefully layered 8ml of blood-PBS mixture on to the ficoll hypaque, keeping the tube in a slanting position. Centrifuged at 2,000 rpm for 30 minutes. The opaque interface containing mononuclear cells was aspirated and transferred into a clean conical centrifuge tube with a pasteur pipette and discarded the upper layer.10ml of PBS solution was added and mixed by inversion. Centrifuged at 1500rpm for 10 minutes and supernatant was discarded. Resuspended pellet with 5ml PBS and centrifuged at 1500rpm for 10 minutes. Repeated the step thrice and resuspended lymphocyte pellet in 500 µl PBS.

*Cytotoxicity studies on normal human lymphocytes lymphocyte viability assay*

The in vitro response of extract ASPE against lymphocyte was studied using lymphocyte viability assay. Lymphocytes were aspirated from the gradient plasma interfaces and washed twice in PBS and the final cell pellets were resuspended in RPMI-1640 medium containing 10% FCS, 100 µl streptomycin and 100 µl fungicide (pH 7.4). Cells were harvested, counted and seeded (5x10^3^ cells/well in 100 µl) in 96 well titre plates (Axygen) and PBS were added to the outer wells (200µl /well). After 24 hours of incubation at 37°C in 5% CO2 to allow cell attachment, media were removed; cultures were treated with varying concentration of extracts diluted with medium. The plates were further incubated for 72 hours. On completion of incubation, with the extracts, media were removed without disturbing the cells and to each well, 100 µl of 5mg/ml stock solution of MTT were added and plates were further incubated for 2 hours in dark at 37°C in a CO2 incubator. 100 µl of lysis buffer was added to each well and the plates were further incubated for 4 hours in dark in a CO2 incubator and absorbance was read using ELISA plate. Three replicates were set up for each concentration.

## Results

*Effect of Annona squamosa seed extracts on DLA and K-562 cells by trypan blue dye exclusion method*

The four extracts (ASPE, ASCH, ASEA and ASME) were assayed by trypan blue for cytotoxicity against the DLA and K-562 cells. Among the extracts the petroleum ether extract (ASPE) showed maximum cytotoxicity of 57 ± 0.8% at a concentration of 800 µg/ml at 12 hrs. Compared to all other extracts ASCH, ASEA and ASME, 33.75±1.2%, 25±0.8% and 50±0.8% respectively at 12 hrs on DLA (Figure l) and 60.75 ±1.5%,45.25±1.7%,28.75±1.7% and 51.25± 0.95% respectively on K-562 cells. (Figure 2). Hence, further studies were conducted using ASPE on adherent cells (KB, A-549, and MCF-7) and suspension cells (K562, DLA, EAC) by MTT assay.

*Effect of Annona squamosa seed extracts on different cells by MTT assay*

In this study different solvent extracts ASPE, ASCH, ASEA and ASME were used to test the cytotoxic activity. When the KB cells treated with four extracts ASPE, ASCH, ASEA and ASME for 400 µg/ml at 24 hours the cytotoxic effect observed were 67.5 ±1.4, 60.5 ± 1.1, 36.5 ± 01.2 and 20.25±0.9 respectively (Figure 3). The A-549 cells treated with four extracts ASPE, ASCH, ASEA and ASME for 400 µg/ml at 24 hours the cytotoxic effect observed were 61.5 ±1.3, 44.5 ± 1.1, 15.5 ± 01.2 and 21.25±0.8 respectively (Figure 4). The MCF-7 cells treated with four extracts ASPE, ASCH, ASEA and ASME for 400 µg/ml at 24 hours the cytotoxic effect observed were 61 ±1.1, 41 ± 1.1, 12±1.25 and 20.25±0.8 respectively (Figure 5). The K- 562 cells treated with four extracts ASPE, ASCH, ASEA and ASME for 400 µg/ml at 24 hours the cytotoxic effect observed were 59.75 ±0.8, 39 ± 1.0, 22 ± 1.1 and 7±0.8 respectively (Figure 6). The DLA cells treated with four extracts ASPE, ASCH, ASEA and ASME for 400 µg/ml at 24 hours the cytotoxic effect observed were 60 ±0.7, 15 ± 0.9, 9 ± 1.1 and 42±0.7 respectively (Figure 7). EAC cells treated with four extracts ASPE, ASCH, ASEA and ASME for 400 µg/ml at 24 hours the cytotoxic effect observed were 62 ±0.9, 9 ± 0.8, 7 ± 1.1 and 13±0.7 respectively (Figure 8). Among the four extracts tested petroleum ether extract (ASPE) showed maximum cytotoxicity (Figure 9). Hence ASPE was used for further studies. The KB cells were treated with ASPE for 12, 24 and 48 hours at 400 µg/ml the cytotoxic effect observed were 63 ±1.2,67.25 ±1.2 and 76 ± 1.3 respectively (Figure 10). The A-549 cells were treated with ASPE for 12, 24 and 48 hours at 400 µg/ml the cytotoxic effect observed were 56.25 ±1.3,61.5 ±1.1, 64.5±1.2 respectively (Figure 11). The MCF- 7 cells were treated with ASPE for 12, 24 and 48 hours at 400 µg/ml the cytotoxic effect observed were 53.5 ±1.1,57.5 ±1.2,60.5 ± 1.3 respectively (Figure 12). The K- 562 cells were treated with ASPE for 12, 24 and 48 hours at 400 µg/ml the cytotoxic effect observed were 53.75 ±1.5, 59.75 ±1.2 and 63.75 ± 1.3 respectively (Figure 13). The DLA cells were treated with ASPE for 12, 24 and 48 hours at 400 µg/ml the cytotoxic effect observed were 56.5 ±1.4, 60.5 ±1.5 and 62.25 ± 1.4 respectively (Figure 14).

The EAC cells were treated with ASPE for 12, 24 and 48 hours at 400 µg/ml the cytotoxic effect observed were 38 ±0.9,62.5 ±1.3 and 64 ± 1.5 respectively (Figure 15). Among the six cell lines tested maximum cytotoxic effect observed was on KB cells 76 ± 1.3.The IC_50_ value of ASPE on KB, A-549, MCF-7, K-562, DLA and EAC were observed as 22.4 µg/ml,

154 µg /ml, 136 µg/ml, 90 µg/ml, 149 µg/ml 98 µlg/ml respectively at 24 hours (Table 1). There were no significant toxicity towards lymphocytes (Figure 16).

## Discussion

Results showed that among the four extracts tested ASPE showed maximum cytotoxic activity. Cell viability assay showed that ASPE could inhibit the proliferation of tumour cells in a concentration and time dependent manner. Hence, we selected ASPE for further studies. In this study petroleum ether showed significant cytotoxicity towards six cell lines and non-significant toxicity towards normal lymphocytes. A survey of the literature data revealed that the antitumour activities of *Annona squamosa* had not been reported earlier in these cell lines. Extracts which exhibited substantial antiproliferative activity represent a source for novel natural anticancer entities. The *Annona squamosa* is traditionally used for the treatment of cardiac problems, antibacterial infection, and antitumor properties. There were earlier reports on ethanolic extracts of leaves and stem to had an anticancerous activity (Saleem et al., 2009). *Annona squamosa* possess antioxidant activity this promotes the anticancer properties (Vikas et al., 2017). Antioxidant promote the anticancer activity. We have studied the cytotoxicity and antiproliferative activity of AS seed extracts active components from natural resources from medicinal plants. For this AS seeds were extracted serially with petroleum ether, chloroform, ethyl acetate, and methanol and each extracts were examined for the cytotoxicity in vitro, on the basis of the direct cytotoxicity of them against six kinds of cultured tumour cell lines, i.e., KB, A-549, MCF-7, K-562, DLA and EAC. In this study, the petroleum ether extracts of the seeds of *Annona squamosa* exhibited marked cytotoxicity against examined tumour cells. Earlier studies showed that organic and aqueous extracts from the seeds of A. squamosa induced apoptosis in MCF-7, breast carcinoma and K- 562, erythrocyte leukemia and COLO-205 colon carcinoma cells. Treatment of these cells with the extract resulted in the induction of reactive oxygen species (ROS) generation and reduces intracellular glutathione levels. In addition, down regulation of BCl-2 and phosphytidyl externalization by Annexin V staining suggested induction of apoptosis. The extracts from *Annona squamosa* also caused significant apoptotic induction in a rat histiocytic tumour cells AK-5 cells (Pardhasaradhi et al., 2004; Pardhasaradhi et al., 2005). Ethanolic extract of *Annona squamosa* showed significant antioxidant activity and anticancerous activity (Bhakuni et al., 1969; Pandey and Barve, 2011). Earlier reports from our laboratory showed cytotoxic effects of *Annona *squamosa on Dalton’s lymphoma cells and HeLa cells (Joy and Remani, 2008). Literatures of many research works prove that every parts of* Annona* squamosa possess medicinal property (Kirtikar and Basu, 1993). Different parts of *Annona* squamosa are used in folkloric medicine for the treatment of various diseases (Suresh et al., 2006). In Ayurveda, fruits are considered as a good tonic, enrich blood, increases muscular strength, cooling, lessens burning sensation and tendency to biliousness, sedative to heart and relieves vomiting (Patel and Kumar, 2008). Plants produce a diverse range of bioactive molecules, making them a rich source of different types of medicines. Higher plants, as sources of medicinal compounds, have continued to play a dominant role in the maintenance of human health since ancient times (Farombi, 2003). Natural products from plants are proven templates for new drug development (Okunade et al., 2004), and have many interesting biological activities. The crushed leaves are sniffed to overcome hysteria and fainting spells they are also applied on ulcer and wounds (Vyas et al., 2012). The present study described the cytotoxic activity of the crude extracts from the seeds of *Annona squamosa*. ASPE is capable of exerting significant cytotoxic effect on cancer cells, sparing normal cells. Our study reports the significant cytotoxic effects of ASPE from *Annona squamosa* on the nasopharyngeal cells, human lung cancer cells, Breast cancer cells, leukemic cells and the murine lymphoma cells. The active principles present in the *Annona squamosa* petroleum ether extract contributing to the cytotoxicity has to be elucidated further.

In conclusion among the four extracts tested ASPE showed maximum cytotoxic activity. Cell viability assay showed that ASPE could inhibit the proliferation of tumour cells in a concentration and time dependent manner. Petroleum ether extract of *Annona squamosa* showed significant cytotoxicity towards six cell lines and non-significant toxicity towards normal lymphocytes. This indicate that *Annona squamosa* seed extract possess compounds that possess anticancer activity on a panel of cancer cell lines can further develop as potent anticancer agents.
